# Diagnosis, prognosis, and management of cryptogenic stroke

**DOI:** 10.12688/f1000research.7384.1

**Published:** 2016-02-12

**Authors:** Cen Zhang, Scott Kasner

**Affiliations:** 1 Department of Neurology, Hospital of the University of Pennsylvania, Philadelphia, Pennsylvania, 19104, USA

**Keywords:** Cryptogenic Stroke, diagnosis of cryptogenic stroke, prognosis of cryptogenic stroke, secondary stroke prevention, Management of cryptogenic stroke

## Abstract

Despite many advances in our understanding of ischemic stroke, cryptogenic strokes (those that do not have a determined etiology) remain a diagnostic and therapeutic challenge. Previous classification approaches to cryptogenic stroke have led to inconsistent definitions, and evidence to determine optimal treatment is scarce. These limitations have prompted international efforts to redefine cryptogenic strokes, leading to more rigorous diagnostic criteria, outcome studies, and new clinical trials. Improvement in our ability to detect paroxysmal atrial fibrillation in patients with cryptogenic stroke has strengthened the idea that these strokes are embolic in nature. Further, better understanding of acute biomarkers has helped to identify otherwise occult mechanisms. Together, these strategies will inform long-term outcomes and shape management.

## Introduction

Cryptogenic stroke refers to stroke of unknown etiology and accounts for approximately 15–40% of all ischemic strokes ^1, 2^. Though cryptogenic stroke seems common, the term lacks specificity and leads to great variability among studies. Moreover, there are no randomized controlled trials to guide long-term treatment. In this review, we will discuss current and emerging diagnostic criteria for cryptogenic stroke, long-term outcomes, and therapeutic options.

## Diagnosis

The diagnosis of cryptogenic stroke has traditionally been based on the exclusion of other well-established causes of stroke ^3^. Three classification systems have frequently been employed to define subtypes or mechanisms of ischemic stroke: the Trial of Org 10172 in Acute Stroke Treatment (TOAST) system, the Causative Classification of Stroke System (CCS), and the Atherosclerosis, Small vessel disease, Cardiac causes, Other, and Dissection (ASCOD) scheme ^4– 6^. None formally define cryptogenic stroke. TOAST includes a category of “stroke of undetermined etiology”, which includes strokes of unknown source despite an extensive evaluation, as well as those with incomplete evaluation and those with more than one identified etiology. CCS offers a category of “undetermined stroke”, which similarly includes subcategories of unknown-cryptogenic embolism, unclassified, and incomplete evaluation. ASCOD has no specific category for stroke of unknown cause but includes them in the category of “other”. While all three systems differ in how unknown or cryptogenic stroke is defined, with different inter-rater reliability, they all require exclusion of other well-established singular etiologies. Notably, these systems do not mandate a minimum set of diagnostic tests, and as a result they will classify several distinct groups as cryptogenic, including patients who had a very extensive evaluation that proved normal, and those with very limited or even no testing that also was unrevealing. Arguably, a thorough evaluation requires brain imaging, with computed tomography (CT) or magnetic resonance imaging (MRI), neurovascular imaging with CT angiography (CTA), MR angiography (MRA) or cervical carotid duplex and transcranial Doppler, cardiac evaluation with echocardiography, and, in select patients, rapid plasmin reagin (RPR), erythrocyte sedimentation rate (ESR), hypercoagulable testing, genetic analysis, or other tests for atypical causes. A broader consensus is required to define the characteristics and criteria for a diagnosis of cryptogenic stroke.

Recent evidence suggesting cryptogenic stroke is likely due to embolic sources has altered these familiar but vague definitions, leading to a new and more rigorously defined term, embolic strokes of undetermined source (ESUS). Introduced by the Cryptogenic Stroke/ESUS International Working Group, this term offers a way to define cryptogenic stroke based on established criteria, rather than due to the lack of an explanation. Diagnostic criteria for ESUS include brain CT or MRI to demonstrate non-lacunar stroke, extracranial and intracranial imaging to exclude ≥50% proximal stenosis, and electrocardiography, echocardiography, and cardiac rhythm monitoring for ≥24 hours to exclude cardioembolic sources ^7^. The ESUS definition likely remains highly heterogeneous, including cardiac abnormalities of uncertain risk (e.g. covert paroxysmal atrial fibrillation [AF], mitral annular calcification, aortic valve disease, or atrial pathology), arteriogenic embolism (e.g. from a nonstenotic ulcerated plaque), paradoxical embolism (e.g. patent foramen ovale or pulmonary arteriovenous malformation), and unknown prothrombotic disorders (e.g. occult malignancy), but provides a useful construct for clinical and research purposes.

Better understanding of the pathophysiology of cryptogenic stroke may also improve its diagnosis and characterization. For example, acute blood biomarkers, including brain natriuretic peptide (BNP), N-terminal proBNP (NT-proBNP), and D-dimer, have emerged as potential aids in determining the underlying etiology of cryptogenic stroke. In a recent meta-analysis of 2834 patients, levels of BNP and NT-proBNP were significantly elevated in patients with cardioembolic stroke, independent of other clinical factors ^8^. In addition, a post-hoc analysis of a subset of participants in the Warfarin vs. Aspirin for Recurrent Stroke Study (WARSS) demonstrated no difference between aspirin and warfarin on the risk of stroke or vascular death when NT-proBNP level was ≤750 pg/mL (hazard ratio [HR] 1.21, p=0.243), but warfarin reduced the risk compared to aspirin when NT-proBNP levels were >750 pg/mL (HR 0.30, p=0.021) ^9^. These studies suggest that acutely elevated levels of BNP in patients with cryptogenic stroke may harbor an underlying or occult cardioembolic mechanism.

Similarly, acutely elevated D-dimer levels after a stroke may implicate a hypercoagulable state secondary to an occult malignancy. A study by Schwarzbach *et al.* comparing 140 patients with cancer and ischemic stroke to 140 age- and sex-matched control patients with stroke alone demonstrated that cancer was associated with a higher prevalence of unidentified strokes (48% vs. 27%, p<0.001) as well as higher levels of D-dimer (6.15 µg/mL vs. 1.39 µg/mL, p<0.001) ^10^. An analysis by Kim *et al.* similarly showed higher levels of D-dimer in patients with cancer and cryptogenic stroke compared to those with cryptogenic stroke without cancer as well as a control group of patients with cancer without stroke ^11^.

## Prognosis

The prognosis of cryptogenic stroke varies, which likely reflects the heterogeneity of the definition as well as the shortage of studies. Several new studies examining long-term outcomes among stroke subtypes, including ESUS, provide comparative data. A retrospective cohort analysis at the Helsinki University Hospital examined recurrent stroke and death risk in a subset of patients referred to as having had an undetermined stroke with an embolic pattern (USEP) ^12^. These patients had embolic lesions on neuroimaging without having completed a full diagnostic evaluation and were further classified into whether or not they met the cryptogenic stroke/ESUS criteria. Among 540 patients with ischemic stroke 23.5% were classified as USEP, and within this group 36.2%, or 8.5% of all patients, met criteria for ESUS. At 21 months, USEP was associated with a higher risk of recurrent stroke compared to both noncardioembolic (HR 2.36, p=0.046) and cardioembolic strokes with known source (HR 1.83, p=0.028). Among the USEP subgroup, there was no difference in risk of recurrent stroke between those who met ESUS criteria versus those who did not.

More recently, Ntaios *et al.* described the long-term outcomes of ESUS patients in the Athens Stroke Registry ^13^. This retrospective analysis included 2731 patients with first ischemic strokes between 1992 and 2011, followed for a mean of 31 months; 10% were diagnosed as ESUS using the cryptogenic stroke/ESUS International Working Group criteria. In this population, the cumulative probability of stroke recurrence in ESUS was similar to cardioembolic strokes (29% vs. 27%) but higher than all other types of noncardioembolic stroke, including large artery atherosclerosis (13%) and lacunar strokes (13%). Notably, there was a higher percentage of ESUS patients with a favorable functional outcome, defined as modified Rankin scale (mRS) ≤2 (62.5%), compared to patients with cardioembolic strokes (32.2%).

## Management

The major challenge in managing cryptogenic stroke is secondary stroke prevention of cryptogenic strokes, specifically in choosing antithrombotic therapy. The use of oral anticoagulation for secondary prevention of cardioembolic strokes is well established, and cryptogenic strokes are being recognized as sharing many features with cardioembolic strokes. However, there are currently no well-founded guidelines for optimal long-term treatment. According to the American Heart Association/American Stroke Association and the American College of Chest Physicians, antiplatelet agents are preferred for noncardioembolic ischemic strokes. A global survey of hospitals in 48 countries found that the vast majority (94%) routinely prescribed antiplatelet therapy for secondary prevention of cryptogenic stroke ^14^, yet there is growing evidence that cryptogenic stroke patients may benefit from anticoagulation. To date, the only randomized trial data comparing the efficacy of anticoagulation to antiplatelet therapy in cryptogenic stroke are derived from post-hoc analyses of the WARSS trial. While the primary analysis of WARSS showed no significant advantage of warfarin compared to aspirin in secondary prevention of noncardioembolic strokes, the subgroup analysis suggested that warfarin was associated with one-third fewer recurrent strokes than aspirin in cryptogenic stroke patients with an embolic appearance, though the result did not reach statistical significance ^15^.

Despite the absence of large, randomized controlled trials, emerging data linking cardiac abnormalities to cryptogenic strokes have shifted management increasingly in favor of anticoagulation. Recent studies show that using prolonged cardiac monitoring devices provides better detection of paroxysmal AF in patients with cryptogenic stroke ^16^. The Cryptogenic Stroke and Underlying AF (CRYSTAL-AF) trial demonstrated that the use of an implantable cardiac monitor increased the rate of AF detection significantly compared to standard monitoring at 6 months (8.9% vs. 1.4%, p<0.001) ^17^. Similarly, the 30-Day Cardiac Event Monitor Belt for Recording AF After a Cerebral Ischemic Event (EMBRACE) trial showed that use of a 30-day loop recorder increases the yield of AF detection in patients diagnosed with cryptogenic stroke (16.1% vs. 3.2%, p<0.001) ^18^. While neither of these studies claimed a causal link between paroxysmal AF and cryptogenic stroke, there was a significantly higher rate of subsequent oral anticoagulation use in the group who underwent prolonged monitoring. In CRYSTAL-AF, anticoagulant use was observed in 10.1% of the intervention group, compared to 4.6% of the control group at 6 months (p=0.04), and 14.7% versus 6% at 12 months (p=0.007). In EMBRACE, anticoagulant use at 90 days was 18.6% in the intervention group compared to 11.1% of the control group (p=0.01). In addition, there was a higher rate of conversion from antiplatelet to anticoagulation in the EMBRACE intervention group compared to the control group (13.6% versus 4.7%, p<0.001). The Asymptomatic Atrial Fibrillation and Stroke Evaluation in Pacemaker Patients and the Atrial Fibrillation Reduction Atrial Pacing Trial (ASSERT) demonstrated that in patients without a prior history of AF, detection of subclinical atrial tachyarrhythmia (AT) lasting at least 6 minutes by pacemakers or implantable cardioverter-defibrillators was correlated with a 2.5-fold increased risk of ischemic stroke (p=0.008) ^19^. Taken together, these studies imply that detection of occult AF in cryptogenic stroke may warrant treatment with anticoagulation. A standard approach in our practice is to monitor patients in whom cardioembolic source is strongly suspected for 30 days using a mobile cardiac outpatient telemetry unit (MCOT). Patients typically remain on single antiplatelet therapy unless AF is detected.

A major area of ongoing uncertainty relates to the minimal duration of AF needed to increase the risk of ischemic stroke and the total burden needed to warrant treatment with anticoagulation ^19, 20^. While the recent advances in technology allow for detection of AF episodes even less than 30 seconds, there are no reliable data demonstrating a clear role for anticoagulation in such circumstances. Thus, how to treat patients in whom short-duration AF is detected after device implantation remains unclear, and is the focus of ongoing trials including Apixaban for the Reduction of Thrombo-Embolism in Patients with Device-Detected Sub-Clinical Atrial Fibrillation (ARTESiA), a prospective study to assess whether anticoagulation reduces risk of stroke and systemic embolism in patients with device-detected subclinical AF ^21^.

Further, the detection of paroxysmal AF does not necessarily provide an etiologic mechanism for cryptogenic stroke. A follow-up study by the ASSERT investigators questioned the causality of subclinical AT and ischemic stroke, noting that arrhythmia detection was temporally not related to the index stroke event ^20^. These findings suggest that paroxysmal AF may be a risk factor or marker for other comorbidities that increase the risk of stroke, rather than the sole or primary etiology. In contrast, Turakhia *et al.* recently established a temporal relationship through a case-crossover analysis of patients with implanted cardiac devices who developed AF and stroke ^22^. Within a subset of patients in whom 120 days of monitoring was available, an episode of AF that was at least 5.5 hours in a day increased the short-term risk of stroke by 4- to 5-fold in the 5 to 10 days after the AF event and decreased over time.

Finally, the role of novel oral anticoagulants in cryptogenic stroke has yet to be determined. Their superior efficacy and improved safety profile compared to warfarin have prompted interest in the prevention of strokes presumably due to embolism, beyond those attributed to nonvalvular AF. Two recently launched randomized controlled trials, Rivaroxaban Versus Aspirin in Secondary Prevention of Stroke and Prevention of Systemic Embolism in Patients With Recent Embolic Stroke of Undetermined Source (NAVIGATE ESUS) and Dabigatran Etexilate for Secondary Stroke Prevention in Patients With Embolic Stroke of Undetermined Source (RE-SPECT ESUS), will assess the efficacy of rivaroxaban and dabigatran, respectively, compared to aspirin in secondary prevention of cryptogenic stroke specifically in ESUS patients.

## Conclusion

Our understanding of cryptogenic strokes has advanced with better technology and ongoing efforts to redefine this category. Newer cardiac monitoring devices continue to improve our ability to detect subclinical AF. Studies are underway to help us understand how to interpret these findings and which treatments improve clinical outcomes. Shifting from cryptogenic stroke to ESUS may create a stronger framework, within which we can provide focused evaluations and uniform criteria for future clinical trials. And, lastly, the completion of NAVIGATE ESUS and RE-SPECT ESUS may provide two new alternatives to our armamentarium of therapeutics.
